# Restoration of Worn Movable Bridge Props with Use of Bronze Claddings

**DOI:** 10.3390/ma11040459

**Published:** 2018-03-21

**Authors:** Ján Viňáš, Marek Vrabeľ, Miroslav Greš, Jakub Brezina, Dušan Sabadka, Gabriel Fedorko, Vieroslav Molnár

**Affiliations:** 1 Faculty of Mechanical Engineering, Technical University of Košice, Mäsiarska 74, 04001 Košice, Slovak; jan.vinas@tuke.sk (J.V.); marek.vrabel@tuke.sk (M.V.); miroslav.gres@tuke.sk (M.G.); jakub.brezina@tuke.sk (J.B.); dusan.sabadka@tuke.sk (D.S.); 2 Technical University of Košice, Letná 9, 04200 Košice, Slovak; gabriel.fedorko@tuke.sk

**Keywords:** cladding, metallographic analysis, hardness, sliding bearing

## Abstract

This article examined the possibility of using CuSn6P claddings in sliding bearing renovation of movable pontoon bridge props. The bronze layer was welded on cylinders of the high-strength steel S355J0WP EN 10155-93, in an inert atmosphere using an automated welding method (gas tungsten arc welding). Pulsed arc welding was used to minimize the effects of heat on the cladding area, while also accounting for the differences in the physical properties of the joined metals. The sliding bearing was created in two layers. The quality of the cladding layer was evaluated by nondestructive and/or destructive tests. The quality of the surface was assessed by visual inspection (visual testing) in accordance with the EN ISO 17637 standard. The quality of the claddings was evaluated by metallographic analysis, performed using light microscopy. The microhardness values of a few weld areas were determined by Vickers tests, performed according to the EN ISO 9015–2 standard. The analyses confirmed that the welding parameters and filler material used resulted in high-quality weld joints with no internal (subsurface) or metallurgical defects.

## 1. Introduction

Nowadays, owing to climatic changes and extreme weather conditions, public safety is increasingly becoming a seasonable problem. During extreme periodic rains, massive economic damage and human losses can occur, owing to the flooding of large residential areas and/or damage to infrastructure, including bridges. While barriers are used to hold back excessive water in stream beds, mobile pilot bridges are employed as civil protection structures for evacuating people and/or restoring food services by providing a fast and reliable connection to the damaged infrastructure. Further, apart from being used for civil protection, mobile bridges are also used by military forces to provide mobility to ground forces. Given these uses of mobile structures such as bridges which require that the structures exhibit good strength and mobility characteristics, they are usually made from high-strength materials, allowing the reduction of particular components of the cross-section to cut their weight. For the functional and movable parts of these bridges, materials that exhibit a high surface resistance against adhesive and/or abrasive wear are generally used. Components such as telescopic props allow for the movement of the individual parts as well as the safe installation of the bridges. Further, as these components can be extended using a hydraulic system, they are also designed to allow for mutual collapse. During collapse, the surfaces of the individual parts are subjected to friction. Thus, to reduce the friction arising from the mutual movement of the moving segments, it is necessary to use sliding bearings. Given the purpose of these props as well as the tribological factors affecting the degradation of the surfaces of the extending cylinders, sliding bearings are used instead of rolling bearings in the positions where the individual moving components come into contact with each other. Sliding bearings are better suited for applications where high loading and low speed is expected. Of the many materials suitable for producing such sliding bearings, CuSn6P-based materials are used widely, given the high strength and desirable sliding- and corrosion-related properties of these materials as well as their low cost. Bronze sliding bearings with cylindrical internal surfaces are deposited by arc welding. The required lifetime of sliding props is approximately 20,000 uplifts. In practice, it is desirable to make the components that are readily replaceable of a softer metal, so that the frictional damage caused to them is limited. This is the general engineering practice in the case of sleeve or journal bearings, whose shaft or journal is made of steel. In these cases, the bearing is made of an alloy of a softer metal, such as tin, copper, lead, aluminium, or bronze [1]. A number of copper alloys are available for use as bearing materials [2,3,4,5]. Pure Cu is not used as a journal bearing material owing its low mechanical strength and hardness. Alloys exhibiting good tribological and mechanical properties give satisfactory results when used for journal bearings [6]. These alloys can generally be divided into five main groups, as follows: copper lead, copper tin (sometimes called tin bronze), leaded bronze, aluminium bronze, and beryllium copper alloys. Tin bronze alloys are characterized by a relatively high hardness and good wear resistance. Guo et al. [7] investigated the microstructure, micro-hardness, and dry friction behaviours of cold-sprayed tin bronze coatings. They reported that the microhardness of sprayed tin bronze coatings (Cu–6 wt % Sn) before heat treatment was approximately 135 HV. Tin bronze castings are used for the movable components of bridges as well as for the turntables for bridges and other structures, such as fixed and expansion bearings that undergo slow or intermittent movement under heavy loads. Bronze alloys, which are also known as tin bronzes, are one of the primary journal bearing materials and are known for their low lead content (less than 0.25%) and high strength [8]. The main function of the tin in these bronzes is to strengthen the alloy. Phosphor bronzes are alloys containing copper, tin, and phosphorous. Phosphor bronzes contain 0.5–11% tin and 0.01–0.35% phosphorous. The phosphorous increases the wear resistance and stiffness of the alloy but decreases its ductility. Phosphor bronzes exhibit superb spring-like qualities, a high fatigue resistance, excellent formability and solderability, and a high corrosion resistance. Further, they allow for twice the maximum pressure than that for tin bronze bearings [9,10,11]. Further, their high strength and wear resistance make them suitable for applications involving high degrees of wear and abrasion. Skroupa et al. [12] investigated CuSn6 weld clad applied onto steel base material by means of the MIG method with regard to its tribological properties. They found out that the friction coefficient is significantly influenced by the number of layers as well as the type of lubricating environment. Moreover, in previous literature [13], authors have evaluated the microstructure and tribological properties of Cu–Sn coating to improve the comprehensive performance of the coating and satisfy the self-lubricating property under special working conditions. In addition, CuSn6P overlays can be used under extreme conditions, including at temperatures of approximately 450 °C. Phosphor bronze alloys are thus high-potential Cu alloys that exhibit a high wear resistance, good machinability, cold workability, and high fatigue and corrosion resistance [14].

## 2. Metallurgical Aspects of Bronze Cladding

**Figure 1:**
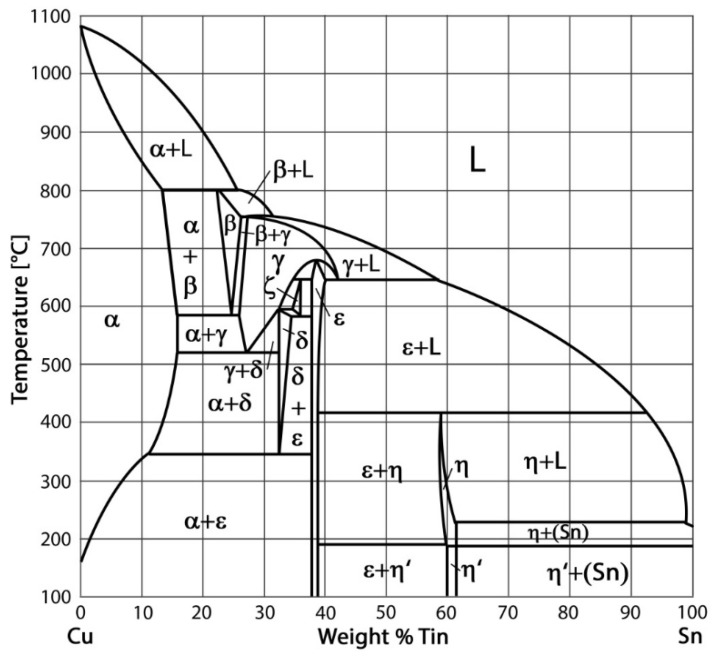
Phase diagram of Cu–Sn binary system.

During Cu–Sn bronze cladding, phase transformations occur as per the binary diagram defined by Hansen [15,16] (see Figure 1). With regard to the soldering process, the α-phase region is the most important one in the diagram for all Sn contents. The maximum solubility of Sn–Cu is approximately 9.1%. Owing to local segregation and inversion segregation, a δ-phase appears in the microstructure of the Cu–Sn alloy for Sn contents of 2–14% during the solidification of the bronze cladding. The α-phase is a substitute solid solution of Sn in Cu with a face-centred cubical lattice and properties similar to those of pure Cu—and, in particular, a plasticity as high as that of pure Cu. On the other hand, the β- and γ-phases have body-centred cubic lattices. Further, the β-phase can undergo a martensitic transformation, after which it transforms into a β′-phase with an orthorhombic lattice. The δ-phase forms a solid solution based on the electron compound, Cu31Sn8 and has a complex cubical lattice. An interesting point to note is that under actual conditions and for temperatures lower than 450 °C, none of the phase transformations in the phase diagram take place. By investigating the microstructures of concrete alloys at ambient temperature, it is possible to see the α-phase or the α-phase in coexistence with a eutectic as well as the eutectic formed by the α + β phases, depend on the Sn content.

During cladding, using alloys with lower Sn contents, phases other than those corresponding to the equilibrium diagram can also form. During the cladding of a Cu alloy on a steel-based material, the Fe in the base material penetrates the weld deposit. Given the low solubility of Fe in Cu, small islands of steel are formed in the weld deposit; this affects the final properties of the weld deposit. Further, so-called brazing cracks are formed in the heat-affected zone (HAZ) in steels during cladding.

### 2.1. Cladding Methods Suitable for Bronze–Steel Applications

Bronze and steel exhibit significantly different physical and mechanical properties, including different melting points, electrical and heat conductivities, hardnesses, and toughnesses. Therefore, special procedures have been designed to join them such that the degree of intermixing of the two weld metals is low. Procedures that involve low heat energy densities are commonly used. During the cladding of bronze on a steel surface, the melting of the weld deposit edges is limited. Moreover, under special conditions, melting does not occur at all [17]. Considering this fact, for claddings made by arc welding using a non-consumable electrode, it is necessary to ensure that most of the electric arc is focused on the copper filler material and/or the cladding metal. Further, it is helpful to form a weld pool, in order to ensure direct contact between the welding materials. Arc welding performed using a consumable electrode requires that the welding arc be right over the weld pool, in order to prevent the melting of the steel base.

### 2.2. Creating and Finishing Cladding Layers

Overlay welding can also be used during the repair of different mechanical parts and components [18,19]. Phosphor bronze is often employed in applications like sleeve bearing and cam followers [20]. Several methods can be used for making the inner cylindrical surface. A few methods that involve arc surfacing are as follows:

- Gas metal arc welding (GMAW)
- Gas tungsten arc welding (GTAW)

Bimetals can also be made by explosion and/or roll cladding. However, conventional arc welding-based methods are primarily used for industrial production, owing to economic factors. The correct cladding technology is chosen based on the results of a theoretical analysis of the weldabilities of the materials in question, according to previous literature [21]. GTAW, which is an automated welding method and is performed in an inert gas atmosphere using a non-consumable electrode, has been used to create the sliding bearings used in pontoon bridge props. The pulse mode is used during the fabrication of the cladding, in order to minimize heat flow to the base material. During pulsed TIG welding, the welding current density is periodically varied between two levels with time; these levels are termed the basic current, Iz, and the pulse current, Ip. The local melting of the base material occurs when the current pulse is applied. The dimensions of the weld pool are primarily determined by the amplitude of Ip and the pulse duration, tp. The pulse shape (rectangular, sinusoidal, trapezoidal, or triangular) also has an effect. Changing these parameters can significantly affect the dimensions of the weld pool [20,21]. For welding and/or cladding, a maximum current of 10 A is used. As mentioned, the amplitude of the current significantly affects the dimensions of the weld pool. During the application of a basic pulse (i.e., when there is a pause between two pulses), there is a decrease in the weld pool dimensions. The function of the current is to ensure continuous arcing together with permanent ionization in the arc area, even during the pauses between the two pulses. The properties of the final cladding weld beads depend on a number of parameters, such as the pulse amplitude, pulse duration, pause between the two pulses, and progressive cladding rate. It is clear that the pause should be longer than the pulse duration (generally tz ≥ 2 tp). If this condition is not met, the weld bath will not solidify fully during the pause—only its dimensions will reduce. This technique is used to ensure that the surfaces of the weld deposits are very smooth and that there is a uniform transition in the base metal. The higher the frequency of the pulses, the higher the welding rate is. In the case of the pulsed-cladding mode, the HAZ is smaller than that for conventional TIG welding. This results in an improvement in the plastic properties in the HAZ and also reduces the susceptibility to cracking. Further, a decrease in the heat input during cladding affects the deformation property [21].

In this study, in order to ensure that the sliding bearings of the bridge props had the desired dimensions and surface quality, machining was performed as a finishing process. Machining copper alloys is considerably easier than machining steels or aluminium alloys of the same strength [22]. The copper–tin alloy, CuSn6P, is used to make heavy-duty plain bearings and hydraulic cylinder bearings, among other types of bearings. A tin bronze alloy with a minimum of 6% Sn is suitable for welding (surfacing and joining). The machinability of lead-free bronzes is significantly lower than that of leaded alloys, owing to the formation of long chips in the former as well as because of their reduced tool life, which results in low stability [23]. Thus, to increase the machinability of lead-free copper-based alloys and to improve the properties of the bearings made using these alloys, Pb is replaced with Bi and Se. Further, other elements such as P and In can be also added in small amounts for this purpose [24]. When machining Cu-based materials with a cemented carbide cutting tool, the clearance must be 8–10°. A large clearance tends to reduce flank wear and makes it easier for the wedge-shaped cutting tool to penetrate the workpiece material. The edge angle, κ_r_, of the tool should be 70–95°. For roughing operations, a tool with a negative cutting edge angle is preferred, while a tool with λ_s_ = 0° is more suitable for light machining or finishing [22]. In terms of the cutting conditions (cutting speed, v_c_; feed rate, f; and depth of cut, ap), it is generally good practice to use the highest practical cutting speed, a relatively light feed, and a moderate depth of cut. Cutting tools made of grade K10 tungsten carbide are recommended for most free-machining brasses, coppers, and copper alloys, while grade K20 is most often employed when milling or making interrupted cuts. During the machining of larger workpieces on lathes, where high cutting speeds are not practicable, the speed should correspond to the lower end of the suggested limits. In machining operations with single-point tools, such as turning, shaping, and planning, the choice of the cutting fluid depends on the material of the cutting tool employed. When using carbide-tipped tools at moderate speeds, cutting may be performed under dry conditions. When bronze and a bronze alumina composite were turned with a K10 tool insert, it was observed that the surface finish decreased with an increase in the cutting speed; the employed cutting speeds were 60–240 m/min [25].

## 3. Materials and Methods

### 3.1. Materials

The chemical composition and mechanical properties of the high-strength construction steel, S 355J0WP EN 10155-93, which is resistant against atmospheric conditions and was used for producing the cylinder to be cladded, are given in Table 1 and Table 2, respectively.

### 3.2. Cladding Parameters

The tin bronze alloy, S Cu 5180 (CuSn6P) EN ISO 24373 with a wire diameter of 1.2 mm was used as the filler material to make the cladding for the sliding bearing. The chemical composition and mechanical properties of the filler material are listed in Table 3. This material is used for reparation of the cast bronzes, welding steels alloyed by copper and other applications. Preheating to 250 °C is strongly recommended for segments with a thickness greater than 5 mm.

**Figure 2:**
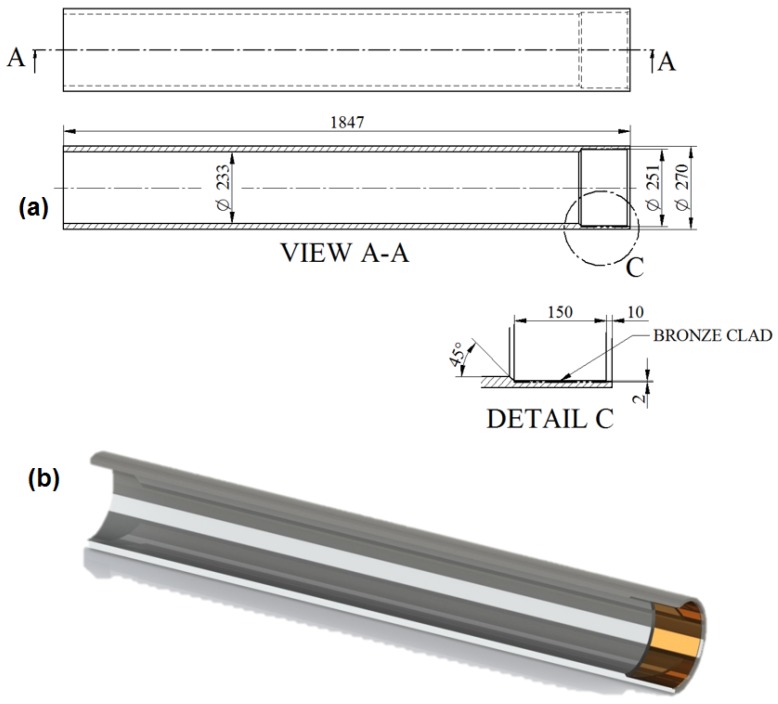
Steel bridge prop with bronze sliding bearing. (**a**) Bridge prop drawing; (**b**) Cross-section of 3D model—created in SolidWorks 2017 CAD software (Dassault Systèmes SOLIDWORKS Corp., MA, USA).

**Figure 3:**
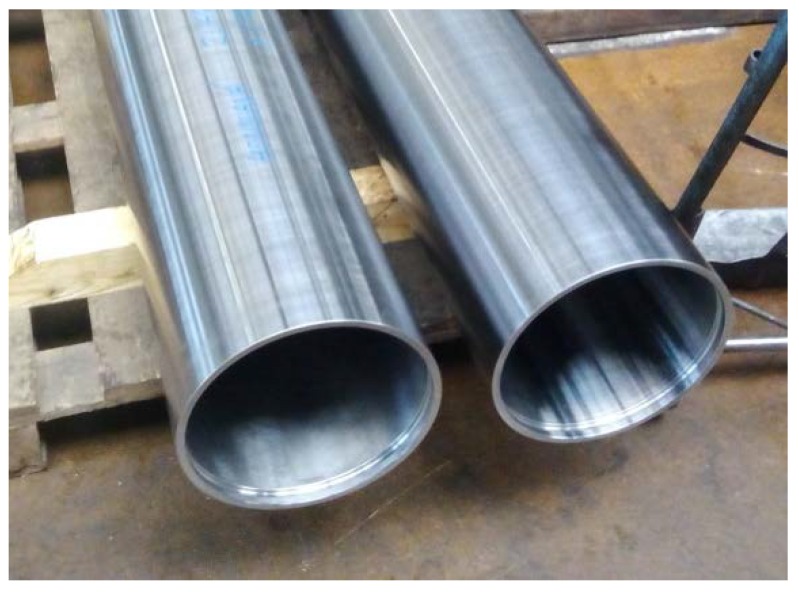
Workpiece steel cylinders before the application of bronze cladding.

The dimensions of the pontoon bridge props are shown in Figure 2a. The cladding deposit was positioned on the internal cylindrical surface as shown in Figure 2b, which had a length of 150 mm from the edge. Images of the props before the cladding procedure are shown in Figure 3.

### 3.3. Cladding Procedure

The cladding was applied by the GTAW method using a welding machine, which allowed the roll rotation parameters to be varied during the cladding process. The cylinder to be cladded was tightened in the independent chucks of a turning lathe that allowed for continuous changes in the rotation of the cylinder. The cladding process was automated. The cladding position used was in accordance with the STN EN ISO 6947-PA standard. This position is the most suitable one for repairing rotary surfaces. The cladding parameters are defined in Table 4. The cladding was of the required length, in spiral form and was applied in two layers, using the shielding gas, Ar 99.999% and an Arc-pulse. The preheating temperature was 330–350 °C, depending on the dimensions and morphological segmentation of the cylinders to be cladded. The first cladding layer was 3.5 mm in thickness. After the application of the cladding, the internal surface was turned to a thickness of 0.8 + 0.2 mm. The interpass temperature was kept at approximately 350 °C.

**Figure 4:**
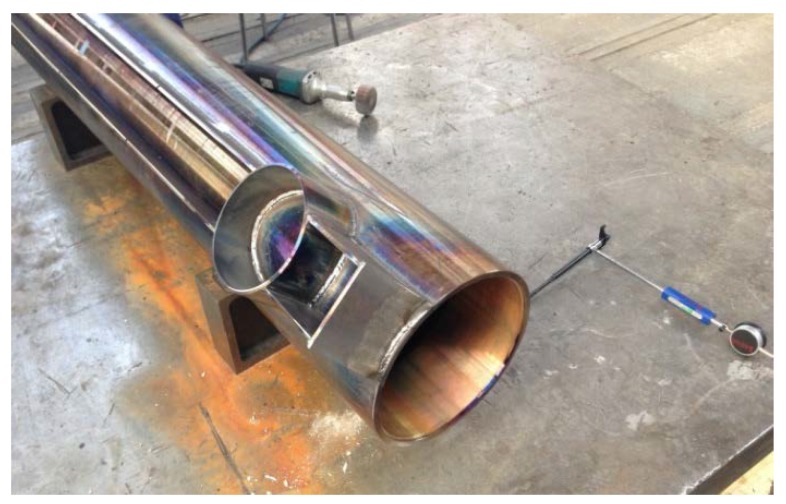
Prop after restoration.

**Figure 5:**
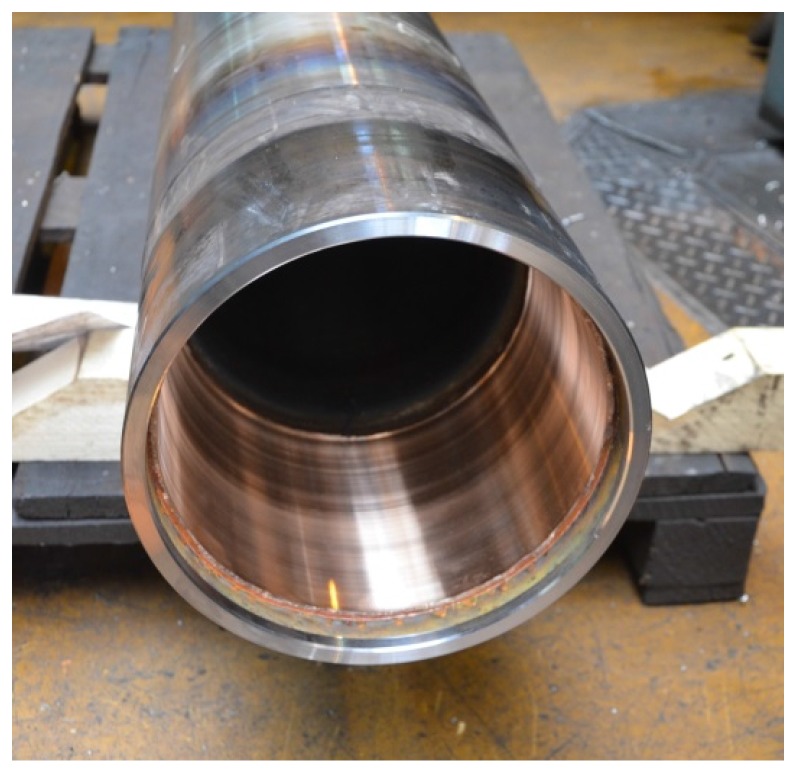
Bronze layer.

The second layer minimized the possibility of the cladding material intermixing with the base material. Further, the chemical composition of the new sliding layer corresponded to that of the filler material. The total required thickness of the cladding was 2 mm. Additional heating was performed at 350–370 °C for 60 min, in order to eliminate the negative internal stresses arising from the differences in the properties of the two alloys used. No additional heat treatment of the cladding was required. The cylinder (length of 150 mm and inner diameter of 251 mm) on which the bronze cladding was to be applied was machined to ensure that its roughness was at the desired level (Ra ≤ 1.6 μm). The machining process was performed on a conventional universal lathe. A 09T304–MPH-coated carbide insert (Korloy) was used to machine the bronze cladding layer to the following geometry: clearance angle of 7°, major edge angle, λs, of 0°, corner radius, rε, of 0.4 mm, and positive rake angle. An S40T–SCLCR–12-type (Korloy) internal tool holder was used for the longitudinal turning operation. Different cutting conditions were employed for the roughing and finishing processes. The depth of cut (ap) was 1.2 mm, the feed rate (f) was 0.2 mm/rev, and the cutting speed (vc) was 40 m/min during the roughing process. For the finishing operation, the machining conditions were as follows: ap of 0.8 mm, f of 0.1 mm/rev, and v_c_ of 60 m/min. Cylindrical turning was performed under dry conditions. The temperature of the workpiece with the cladding layer before the machining process was approximately 300 °C. Both roughing and finishing were performed in a single pass of the cutting tool under the different machining conditions mentioned above. After the completion of the roughing operation, the second cladding layer was applied; this layer was machined to the required thickness of 2 mm during the finishing process. The telescopic props with the bronze claddings are shown in Figure 4 and Figure 5.

A metallographic analysis of the cladding microstructure was performed in accordance with European Technical Standard EN ISO 17639 using a light microscope (Olympus BXFM, Olympus Europa SE & Co. KG, Hamburg, Germany. In order to elucidate the differences between the two alloys, the microstructure of the steel, S355J0WP, was analyzed first. To visualize the microstructure, the steel was etched with Nital (3% HNO_3_). The bronze cladding was etched with an etching agent with the following chemical composition: 10 g FeCl_3_ + 30 mL HCl + 120 mL H_2_O.

The microhardness values of the materials were determined using a semi-automated microhardness tester (Shimadzu HMV, Shimadzu, Kyoto, Japan) using transversal cross-sections in accordance with the STN EN ISO 9015–2 standard. The microhardness was also measured through the cladding—in the HAZ, and in the base material. A pyramidal diamond indenter with a vertex angle of 136° was used. A load force, Fz, of 980.7 mN (=0.9807 N/100 g) was applied for 15 s on the test sample.

## 4. Results

**Figure 6:**
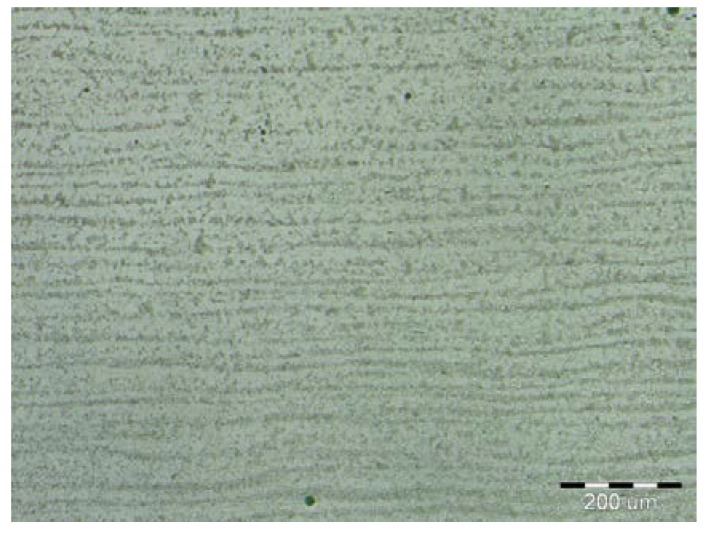
Banded fine-grained microstructure of a base material.

**Figure 7:**
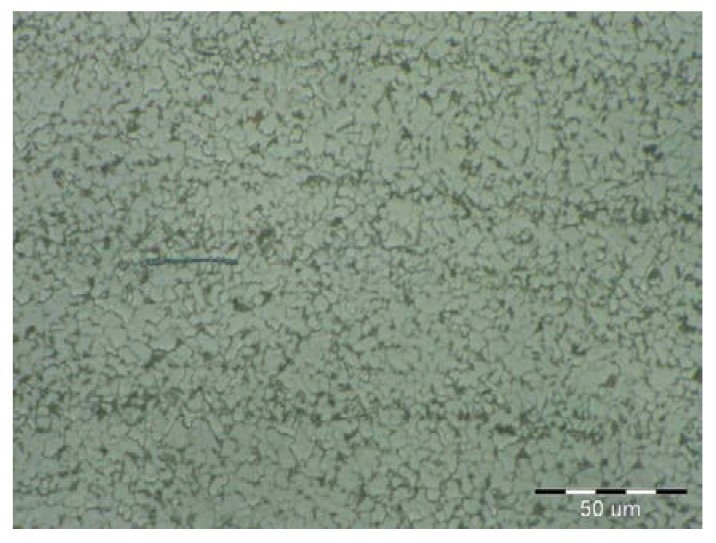
Magnified view of microstructure of base material.

**Figure 8:**
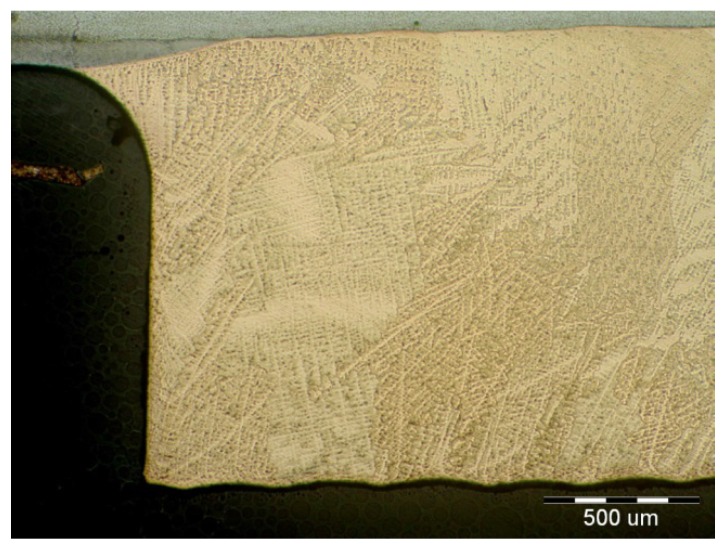
Dendritic microstructure of CuSn6P cladding.

**Figure 9:**
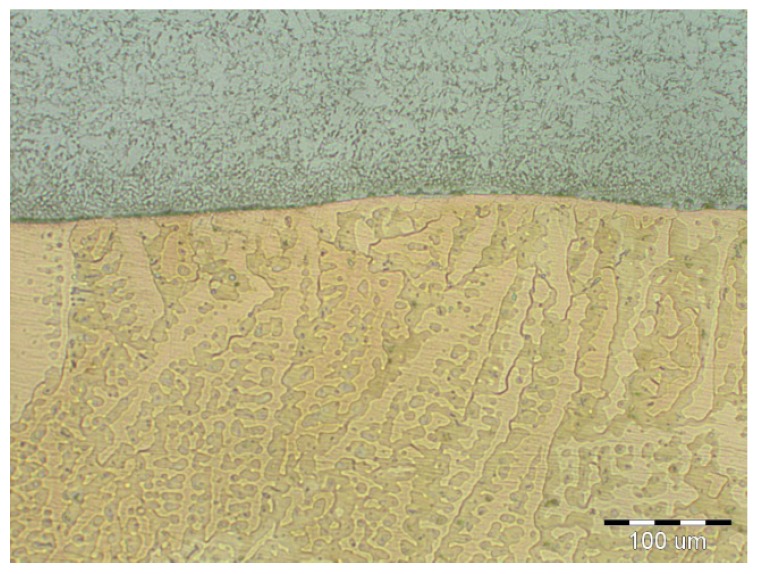
Interface between cladding and BM (base material).

**Figure 10:**
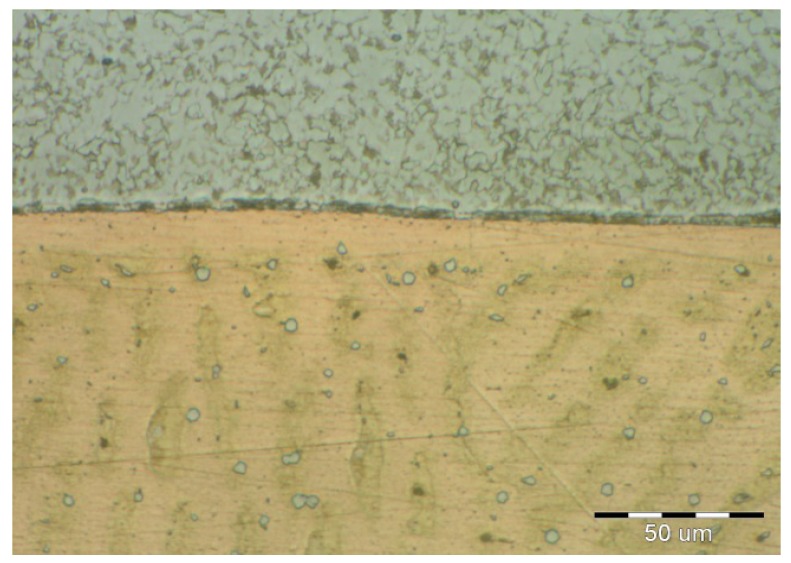
Magnified view of interface between cladding and BM (base material).

**Figure 11:**
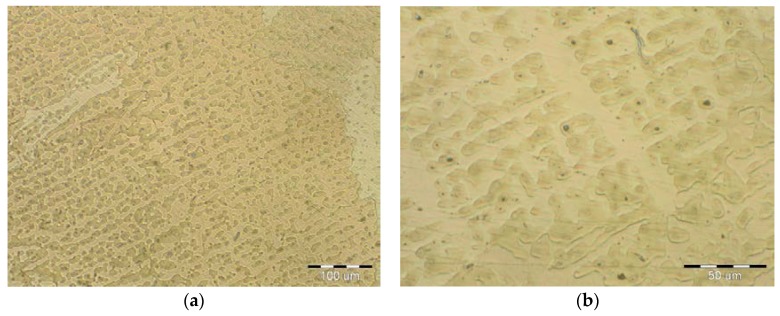
Microstructure of the first-pass layer: (**a**) magnification 200×; (**b**) magnification 500×.

On visual inspection, using a magnifier and an endoscope, no surface defects were found on the cladding. Further, using the reflection method, which involved the use of an Epoch 3 device, no internal defects were detected in the cladding. Finally, based on nondestructive tests, the cladding was confirmed to meet the requirements for level “B” of the European Standard EN ISO 5817. The results of the metallographic analysis are shown in Figure 6, Figure 7, Figure 8, Figure 9, Figure 10 and Figure 11.

The microstructure of the S355J0WP steel base consisted of fine grains (see Figure 6). Further, Figure 7 shows that it consisted of the following microstructural components: ferrite and pearlite. Globular inclusions have been detected previously in microstructure of this steel. However, they were not observed in the current study. A recrystallization region was detected in the narrow HAZ, which corresponded to the short-duration heating of the steel to temperatures of 550–720 °C. The recrystallized grains in this region were reflective of the heat treatment. The globularity of the pearlite phase was characteristic of a heat treatment at temperatures of Ac1 (temperature at which austenite begins to form) to Ac3 (temperature at which ferrite completes its transformation into austenite). The morphology of the ferrite phase was polyhedric, despite the fact that it did not undergo transformation. For the range of temperatures used for the normalization annealing treatment, the ferrite and pearlite phases underwent a transformation.

It can be seen from Figure 8 that a flat interface was formed between the base material and the CuSn6P cladding. This interface was similar to a diffusion joint. Thus, the weld joint can be referred to as a diffusion joint. The degree of intermixing of the base material with the cladding was very low, owing to the differences in the melting points between the two materials (melting point of Cu ≈ 950 °C). The HAZ was narrow and not greater than 0.4 mm in size. The microstructure of the bronze cladding exhibited a typical dendritic structure. Further, the individual dendrite blocks were large.

The microstructural images shown in Figure 9, Figure 10 and Figure 11 confirmed that the cladding parameters chosen were the correct ones, since no intermixing of the cladding and base material was observed. Further, no internal defects such as pores and cavities were seen in the claddings. Finally, the dendritic morphology was crystalline.

The microstructure of the cladding after the first-pass is shown in Figure 11. Moreover, the characteristic arrangement of the bronze dendrites can be seen as well. In the microstructure of the cladding cover layer, both the β-phase and the γ-phase can be seen. The β- and γ-phases have bulk-centered K8 lattices. The β-phase underwent a martensitic transformation. Neither liquation nor segregation were detected between the two cladding layers. These would have been evidenced by interdendritic regions and would have resulted eventually in crystallization detects between the blocks and/or dendrites. Thus, it can be stated that the correct voltage was used for the cladding process, since the voltage is the main factor affecting the thickness uniformity of the cladding layer.

The microstructure of bronze claddings can be dendritic or polyhedric (monophasic) in the transition zones. The dendritic structures formed by the α-phase and the eutectoid precipitates are shown in Figure 11. Only a few small areas with a martensite phase were detected during the analysis. However, oxide inclusions such as CuO and complex oxides (Cu, Fe O) were detected in small amounts. That the oxides were present in small amounts confirmed that the inert gas used provided suitable protection.

**Figure 12:**
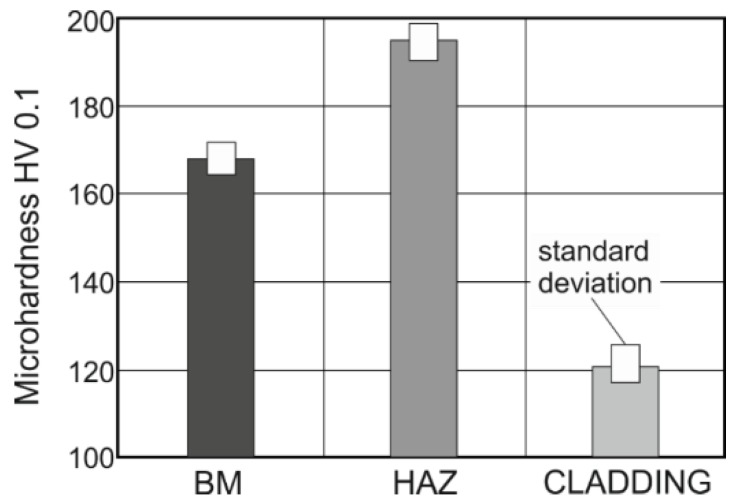
Microhardness (HV 0.1) values measured in different regions (BM: base material, HAZ: heat-affected zone).

The microhardness values were measured in accordance with the European standard, EN ISO 9015–2. The results are shown in terms of average values in Figure 12.

The minimum average microhardness of the cladding alloy CuSn6P was 120 HV 0.1, while that of the base material was 169 HV 0.1. Further, the maximum average value of the HAZ was 194 HV 0.1. These values are in keeping with the chemical compositions of the corresponding materials and their microstructures and how they are affected by heat during the cladding process.

## 5. Conclusions

A procedure for applying bronze claddings for restoring pontoon bridge props was proposed in this study, and the quality of the bronze claddings was analyzed. The following are the key findings of the study:

- Claddings are suitable for protecting the internal functional surfaces of supports that must exhibit good tribological properties, such as a low friction index and high resistance against adhesive wear and corrosion. The functional layers are tightly interconnected with the underlying base material (steel) through so-called diffusion welds. The connections formed between the two materials reduce the production costs of telescopic props. If the cylinder is monolithic and made of bronze (specific weight of CuSn is 8800 kg/m^3^) and/or structural steel and uses a set of ball bearings, it will result in a structure with a high thickness and increased weight.
- Making a bimetal cladding of CuSn6P and steel requires complete optimization of the cladding process. The most important parameter of the cladding process is the heat input, which can be minimized by using the GMAW and GTAW methods and a pulsed welding arc. The cold metal transfer method is also a promising one.
- The formation of microcracks in the transition region between the base and cladding materials is a risk associated with arc cladding. The microcracks can be intergranular with a transversal direction or have a longitudinal orientation with respect to the cladding direction. However, transversal granular cracks are rarely observed in bimetal claddings formed by arc welding.
- GTAW has been confirmed to be a suitable method for the production of bronze CuSn6P claddings on the steel base material with regard to microcrack formation. No microcracks were detected on the interface between the base material and overlay within all tested samples, as shown in Figure 8, Figure 9, Figure 10 and Figure 11.
- The hardness of cladding is about 30% lower than the hardness of the base material (169 HV) while the highest hardness (194 HV) was measured in HAZ.
- The employed shielding gas, Ar 99.999% at rate of 16 L/min, proved to be an eligible protective welding atmosphere because no undesirable oxides were identified in the weld metal.
- Both the β-phase and the γ-phase were observed in the microstructure of the cladding cover layer after the first-pass.
- The quality of the claddings and diffusion at the junction are affected by the inert gas used. The gas purity, volume, and flow pattern depend on the nozzle geometry. The use of improper parameters can result in the local formation of CuO_2_ particles in the cladding–base material transition zone. These particles adversely affect the strength of the weld joints.

## Figures and Tables

**Table 1:** Chemical composition of steel S 355J0WP (wt %).

C	Mn	Si	Cr	Cu
0.10%	0.84%	0.61%	1.11%	0.45%
N	Ni	P	S	Fe
0.02%	0.4%	0.06%	0.02%	Res.

**Table 2:** Mechanical properties of steel S 355J0WP.

Tensile Strength Rm (MPa)	Yield Strength Re (MPa)	Expansion A5 (%)	Impact Energy (J) (0 °C)	Hardness HV
490–630	≥345	≥22	≥27	155

**Table 3:** Chemical composition of S Cu 5180A—EN ISO 24373 and its mechanical properties (GTAW welding wire ER CuSn-A AWS A-5.7).

Sn (%)	P (%)	Cu (%)	Tensile Strength Rm (MPa)	Expansion A5 (%)	Impact Energy (J) (20 °C)	Hardness HB
6.3	0.22	Res.	≥260	≥30	≥32	80–60

**Table 4:** Parameters used during the cladding process.

Welding Current (A)	Open Arc Voltage (V)	Travel Speed (cm/min)	Polarity	Travel Angle (°)	Wire Feed Speed (m/min)
145	22	42	DC	15	0.8
